# A Study Comparing Acute Toxicities of Cetuximab and Cisplatin in Patients Undergoing Definitive Chemoradiation With Intensity-Modulated Radiotherapy for Locally Advanced Carcinoma Head and Neck

**DOI:** 10.7759/cureus.16505

**Published:** 2021-07-20

**Authors:** Shruthi Venkateshulu, Kiran Kumar BR

**Affiliations:** 1 Radiation Oncology, VTSM Peripheral Cancer Centre, Kalaburgi, IND; 2 Radiation Oncology, Bangalore Medical college and Research Institute, Bangalore, IND

**Keywords:** cetuximab, cisplatin, hnscc, head and neck cancer, chemotherapy

## Abstract

Purpose

To study the acute toxicities of weekly cetuximab used concurrently with intensity-modulated radiotherapy (IMRT) versus weekly cisplatin with concurrent IMRT in locoregionally advanced head and neck squamous cell carcinoma (HNSCC).

Materials and methods

Fifty eligible patients were randomly assigned to study group (cetuximab group) and control group (cisplatin group) to receive cetuximab 400 mg/m^2^ intravenously one week prior to the start of RT followed by 250 mg/m^2^/week, or weekly cisplatin intravenously 40 mg/m^2^, during RT. RT dose received was 70Gy in 35 fractions with 2Gy/fraction in both the arms with IMRT technique. Patients are reviewed every week and Patients were evaluated for acute toxicities according to radiation therapy oncology group (RTOG) toxicity scoring criteria and toxicities grade was reported.

Results

The incidence of acute toxicities like oral mucositis, dysphagia, nausea, vomiting, and leukopenia were much less in cetuximab arm and statistically significant difference was observed as compared to cisplatin arm. and no major systemic toxicities were encountered in the cetuximab arm.

Conclusion

Weekly cetuximab with concurrent radiotherapy in locally advanced HNSCC is a promising regimen with well tolerable toxicity profile. Larger prospective randomized studies with a longer duration of follow-up with direct comparison of both the regimens are needed for strong evaluation of efficacy and toxicity profile.

## Introduction

Head and neck squamous cell cancer (HNSCC) is one of the most common malignancies worldwide, with an incidence of 800,000 new cases annually. [1]. In India, it constitutes almost one-third of all cancer cases [2]. Mortality in India due to head and neck cancer is at least half the incidence due to its late presentation for treatment (stage III - 39%, stage IV- 23%) [3]. Surgery combined with radiotherapy (RT) with or without chemotherapy is the preferred treatment in locoregionally advanced HNSCC. However, in patients with unresectable disease, definitive RT remains the treatment of choice [4]. The standard treatment for patients, who are not suitable for surgery is concurrent Chemoradiation. There are two common treatment strategies are concurrent cisplatin, or cetuximab with RT. Platinum-based chemoradiotherapy is the standard approach for locally advanced HNSCC in many countries.

Many large phase 3 trials and meta-analyses have shown that concurrent cisplatin with radiotherapy (CTRT) improves overall survival (OS) compared with RT alone. However, CTRT leads to numerous toxicities [5-7]. According to updated meta-analysis of chemotherapy in head and neck cancer (MACH-NC) metanalysis, the addition of concomitant cisplatin to RT improves outcome, with an absolute gain in overall survival (OS) of 6.5% at five years [8]. Cisplatin administered intravenously at a dose of 100 mg/m^2^ every third week is the most established regimen, there are several other schedules, mainly weekly low-dose regimens, have also been reported [9-11].

As patients may develop serious toxicity of cisplatin, that could affect their quality of life (QOL). Radiotherapy can induce the expression of epidermal growth factor receptor (EGFR) in HNSCC, leading to resistance [12]. Cetuximab, a monoclonal antibody that targets the EGFR, is the first targeted treatment that shows therapeutic efficacy in HNSCC and may help to overcome this resistance. Cetuximab, has been approved by the Food and Drug Administration agency (FDA), for use in patients with locally advanced HNSCC [13]. Similarly, some phase 3 trials have shown that concurrent cetuximab with radiation therapy improves OS, LRC, and the quality of life compared with RT alone [14]. Updated results with five-year survival reported by Bonner et al showed that RT combined with the epidermal growth factor receptor (EGFR) antibody cetuximab showed an absolute benefit of 9.2% (five-year survival of 45.6% in the cetuximab plus RT group versus 36.4% in the radiotherapy alone group) which is similar to that of concurrent cisplatin [15]. Cetuximab has been increasingly used to treat patients who concern about the toxicity of platinum-based regimens. Cetuximab appears to have less toxicity profile than high-dose cisplatin. In the phase III study of cetuximab and radiotherapy for locally advanced non-operative HNSCC, 93% of patients received the prescribed cetuximab dose, which compares very favourably to the compliance rate of high dose cisplatin in RTOG 95-01 (61%) [16].

Recently, two randomized trials revealed that cetuximab with radiotherapy was inferior to cisplatin with RT for patients with human papillomavirus (HPV) positive oropharyngeal carcinoma [17,18]. However, except for its highly selected group, there are no robust trials with direct comparison of the efficacy of weekly cetuximab against cisplatin with concurrent radiotherapy in locally advanced HNSCC. Most of the data published in various journals are of retrospective studies. which comprises the majority in HNSCC. Several studies also suggest that EGFR inhibition might be more effective in HPV Negative cancers than in HPV-positive cancers [19,20]. Hence, we conducted a prospective study comparing acute toxicities of cetuximab concurrent with IMRT versus cisplatin concurrent with IMRT in patients with locally advanced HNSCC.

## Materials and methods

It was a hospital-based prospective randomized comparative study.

Patients aged above 18 years with previously untreated locally advanced HNSCC of the stage III-IV according to American Joint Committee on Cancer Staging (AJCC) TNM classification, 7th edition, without distant metastases, Performance status of 0-2 according to ECOG and who are eligible for definitive RT were recruited into the study. Patients previously treated with surgery, chemotherapy or radiotherapy and Patients with severe cardiac illness, previous malignancies, poor performance status (ECOG 3 and 4), pregnancy and lactating females were excluded (Table 1 describes the patient and tumor characteristics).

**Table 1:** Patient and tumour characteristics. AJCC: American Joint Committee on Cancer Staging.

Gender	Control arm (N = 25)		Study arm (N = 25)	
	No.	%	No.	%
Male	24	96	23	92
Female	1	4	2	8
Primary site				
Oral cavity	1	4	2	8
Oropharynx	13	52	11	44
Hypopharynx	4	16	6	24
Larynx	7	28	5	20
Nasopharynx	0	0	1	4
Tumour stage				
T1	5	20	0	0
T2	16	64	14	56
T3	3	12	9	36
T4	1	4	2	8
Nodal stage				
N0	3	12	3	12
N1	3	12	5	20
N2	19	76	17	68
N3	0	0	0	0
AJCC stage				
III	5	20	7	28
IVA	20	80	18	72

Fifty biopsy-proven cases of locally advanced HNSCC were taken into the study, after taking the written informed consent. All the patients underwent a detailed history and a general and systemic examination, as well as laboratory tests like complete blood count, kidney function test, liver function test, chest radiographs, ultrasonography abdomen, echocardiogram (ECG), complete ENT evaluation, CECT head & neck. All the patients were randomized to study arm (cetuximab) and control arm (cisplatin) with 25 patients in each arm.

Both the arms were treated with a definitive RT delivered by linear accelerator with IMRT with a dose of 70Gy in 35 Fractions, five days in a week with 2Gy per fraction. Control arm received concurrent cisplatin 40 mg/m^2^ intravenously one-hour infusion with full hydration and supportive medications four to six hours before radiation, repeated weekly for five cycles. Study arm received concurrent cetuximab 400 mg/m^2^ as loading dose over 120 mins infusion, one week prior to radiotherapy followed by weekly dose of 250 mg/m^2^ intravenously one-hour infusion with prior premedication.

Patients are reviewed every week and evaluated for toxicities according to radiotherapy oncology group (RTOG) acute radiation toxicity scoring criteria [21] and Common Terminology Criteria for Adverse Events (CTCAE) Version 4.0 [22] and toxicities grade was reported.

Data analysis was performed with Statistical Package for Social Sciences (SPSS), version 21.0 (IBM Corp., Armonk, NY) statistical package. We have used chi-square test for the analysis. The probability value P < 0.05 was considered significant.

## Results

Fifty patients with locally advanced HNSCC (AJCC 7th edition classification stage III/IV) were entered into the study after taking written informed consent. The patients were divided into two groups Study arm and Control arm with 25 patients in each group by online computer-generated randomization.

The commonest histopathology was moderately differentiated squamous cell carcinoma, with 20 patients in the study group and 19 patients in the control group. Twelve patients (seven in study arm and five in control arm) were of stage III and 38 patients (18 in study group and 20 in control arm) were of stage IVA according to AJCC staging system. Forty-four patients had Eastern Cooperative Oncology Group (ECOG) performance status 0 (21 in study group and 23 in control group) and six patients had ECOG performance status 1 (four in study group and two in control group) (Table 2).

**Table 2:** Performance status and histology distribution of patients. ECOG: Eastern Cooperative Oncology Group.

ECOG performance status	Control group (N = 25)		Study group (N = 25)	
	No	%	No	%
0	23	92	21	84
1	2	8	4	16
Histology				
Well differentiated squamous cell carcinoma	4	16	3	12
Moderately differentiated squamous cell carcinoma	19	76	20	80
Poorly differentiated squamous cell carcinoma	2	8	2	8

The chief adverse effect both in the study group and the control group was acute oral mucositis. The incidence of grade 4 mucositis was more in the control group, i.e., four patients as compared to study group, i.e., one patient. Even grade 3 mucositis was more in the control group i.e 15 patients as compared to the study group, i.e., 10 patients. So significant difference in the incidence of grade III & grade IV mucositis was observed and was more in the control group as compared to the study group. Most of the patients developing grade 3 or 4 mucositis needed dietary modification in the form of liquid diet, and those developing severe mucositis were managed with intravenous fluids, steroids and analgesics (Table 3).

**Table 3:** Incidence of dermatitis, acute mucositis, dysphagia, nausea and vomiting, leukopenia.

Dermatitis grade	Control group (N = 25)		Study group (N = 25)	
	No	%	No	%
I	7	28	8	32
II	15	60	4	16
III	3	12	13	52
IV	0	0	0	0
Acute mucositis				
II	6	24	14	56
III	15	60	10	40
IV	4	16	1	4
Dysphagia				
Grade 1	6	24	8	32
Grade 2	12	48	14	56
Grade 3	4	16	3	12
Grade 4	3	12	0	0
Nausea and vomiting				
Grade 1	15	60	19	76
Grade 2	9	36	6	24
Grade 3	1	4	0	0
Grade 4	0	0	0	0
Leukopenia				
Grade 0	11	44	21	84
Grade 1	0	0	3	12
Grade 2	12	48	1	8
Grade 3	2	8	0	0

Grade 4 & 3 dysphagia was seen in three patients and four patients, respectively, in the control group, whereas only three patients had grade 3 dysphagia in the study group and none of them had grade 4 dysphagia. The incidence of nausea and vomiting in the study group was grade 1 (19 patients), grade 2 (six patients). None of them had grade 3 & grade 4 vomiting. Whereas one patient had grade 3 nausea & vomiting in the control group and six patients had grade 2, and 15 patients had grade 1 nausea & vomiting (Table 3).

The incidence of grade 3 dermatitis was more (13 patients) in the study group as compared to control group (three patients). None of the patients developed grade 4 dermatitis. The incidence of acneiform rash was also more in the study group consisting of five patients with grade 1 , 10 patients with grade 2, three patients with grade 3, one patient with grade 4 reaction. Whereas only three patients had grade 1, and two patients had grade 2 acneiform rash in the control group. The incidence of infusion reactions was more in the study group as compared to control group, one patient had grade 1 infusion reaction, three patients had grade 2 infusion reaction, and one patient had grade 3 infusion reaction in the study group whereas two patients had grade 1 and one patient had grade 2 infusion reaction in the control group. One patient initially enrolled in the study group had severe grade 4 infusion reaction, so the drug was stopped and was eliminated from the study (Table 4).

**Table 4:** Incidence of acneiform rash and infusion reaction.

Study group grade	Control group				Study group			
	I	II	III	IV	I	II	III	IV
Acneiform rash	3	2	0	0	5	10	3	1
Infusion reaction	2	1	0	0	1	3	1	0

Two patients in the study group, four patients in the control group developed grade 1 anaemia, but no significant difference was found (Table 5). Fourteen patients had leukopenia (12 patients had grade 2 and two patients had grade 3) in the control group whereas only one patient had grade 2 leukopenia and three patients had grade 1 leukopenia in the study group, so a significant difference was observed with respect to leukopenia (more in the control group as compared to study group).

**Table 5:** Incidence of anaemia.

Anaemia grade	Control group		Study group	
	No	%	No	%
0	21	84	23	92
1	4	16	2	8

## Discussion

Among various cancers, cancer of the head and neck region is rampant in our developing nation. The problem can be reduced by strict anti-tobacco laws and increasing awareness among people. But at present only answer to the patient population is early detection followed by standard concurrent chemo-radiation (CTRT) as compared to only surgery or surgery and radiotherapy or induction chemotherapy followed by radiotherapy or even hyper-fractionated radiotherapy which has been proved by various randomized trials in the literature It has become clear that use of chemotherapy concurrent with radiation therapy improves both locoregional control and overall survival.

CTRT and bio-radiotherapy (BRT) are both the standard of treatment for patients with locoregionally advanced HNSCC who are not suitable for Surgery. Since there were no randomized phase 3 trials to compare these two regimens for a long time, the opinion that BRT was comparable to CRT has been challenged. Few clinical studies and metanalyses that have addressed this issue have conflicting conclusions. Fausto Petrelli et al conducted a meta-analysis to evaluate the efficacy of platinum-based chemoradiotherapy compared with cetuximab-based bio-radiotherapy in locally advanced HNSCC [23] and they found that cisplatin had better overall survival (OS) and progression-free survival (PFS). However, the risk ratio was defined as the primary measurement of treatment outcome in this study, but the outcome of time-to-event was not considered.

ARTSCAN III, a randomized phase III study conducted by Gebre-Medhin et al. compared chemoradiotherapy with cisplatin and cetuximab in patients with locally advanced HNSCC. Eligible patients in this study received either intravenous cetuximab or weekly intravenous cisplatin 40 mg/m2 along with radiation therapy. Primary endpoint of this study was overall survival (OS). Secondary endpoints were locoregional control, local control with dose-escalated RT, pattern of failure, and adverse effects. Study was prematurely closed after an unplanned interim analysis when 298 patients had been randomly assigned. At three years, OS was 88% and 78% in the cisplatin and cetuximab groups, respectively. The cumulative incidence of locoregional failures at three years was 23% compared with 9% in the cetuximab versus the cisplatin group. They concluded that Cetuximab is inferior to cisplatin regarding locoregional control for concomitant treatment with RT in patients with locoregionally advanced HNSCC and suggested for additional studies to identify possible subgroups that still may benefit from concomitant cetuximab treatment [24].

Our study was intended to compare anti-epidermal growth factor receptor (EGFR) monoclonal antibody cetuximab with concurrent radiotherapy versus concurrent chemoradiation with weekly cisplatin in loc-regionally advanced HNSCC. In this study, we have assessed the acute toxicities with cetuximab versus cisplatin along with IMRT in patients with locally advanced HNSCC.

Grade 3 & 4 mucositis (60% & 16%), grade 3 & 4 dysphagia (16% & 12%), grade 3 nausea & vomiting (4%), were more in the cisplatin arm which are also demonstrated in various other studies in the literature, and the toxicity rates of the same are comparable to a study by Dimri et al [25], where grade-III/IV mucositis was seen in 58% & 9% respectively, and grade III emesis-3%, the results are also comparable to a study by Kang et al. [26], in which grade 3-4 adverse events included mucositis (82.9%), dysphagia (8.6%).

Dermatitis and acneiform rash were more in the cetuximab arm which are comparable to the other studies by Ye et al. [27], In another study by Koutcher et al. [28], and also in a study by Levy et al. [29], which also showed greater skin toxicities and acneiform rash. In cetuximab arm, grade 3 dermatitis was seen in 13 patients (52%) & none had grade 4 dermatitis, which is comparable, to a study by Agarwal et al. [30] in which grade 3 dermatitis was seen in 40.5%of patients. In cisplatin arm grade 3 dermatitis was seen in 3 patients (12%) and none had grade 4 dermatitis, which is comparable to a study by Dimri et al. [25] in which grade 3 and 4 dermatitis was 10% and 1%, respectively.

Infusion reactions were also more in cetuximab arm, one patient (4%) had grade 3 infusion reaction, four patients (one had grade 1, three had grade 2 infusion reactions) which was comparable to a study by Bonner et al. [15] in which 3% patients had grade 3 infusion reaction. Incidence of grade 3 Leukopenia were more in the cisplatin arm with 8% (two patients), respectively.

The incidence of acute toxicities like oral mucositis, dysphagia, nausea, vomiting, and leukopenia were much less in cetuximab arm and statistically significant difference was observed as compared to cisplatin arm. Though the incidence of dermatitis, acneiform rash, infusion reactions were more in the cetuximab arm, they were comparable to that of cisplatin arm and could be manageable with simple measures and supportive treatment, and no major systemic toxicities were encountered in the cetuximab arm, hence we conclude that weekly cetuximab with concurrent radiotherapy in locally advanced HNSCC is a promising regimen with similar efficacy and well tolerable toxicity profile.

As the sample size was less in our study, and the follow-up period of only six months and these are preliminary results, larger prospective randomized studies with longer duration of follow up with direct comparison of both the regimens are needed for strong evaluation of efficacy and to draw inferences about the late toxicities and also loco-regional control (LRC), disease-free-survival (DFS) and OS, which are both the standard of care in locally advanced HNSCC in the current era.

## Conclusions

Weekly cetuximab with concurrent radiotherapy in locally advanced HNSCC is a promising regimen with well tolerable toxicity profile. Compared to cisplatin, the cetuximab has much less acute toxicity profile. Larger prospective randomized studies with a longer duration of follow-up with direct comparison of both the regimens are needed for strong evaluation of efficacy and toxicity profile.
